# Ropeginterferon alfa-2b treatment in a young patient with multi-refractory polycythemia vera and double JAK2 gene mutation: a case report

**DOI:** 10.3389/fonc.2023.1338417

**Published:** 2024-01-09

**Authors:** Silvio Ligia, Emilia Scalzulli, Ida Carmosino, Giovanna Palumbo, Maria Chiara Molinari, Rebecca Poggiali, Alessandro Costa, Maria Laura Bisegna, Maurizio Martelli, Massimo Breccia

**Affiliations:** Hematology, Department of Translational and Precision Medicine, Azienda Policlinico Umberto I - Sapienza University, Rome, Italy

**Keywords:** case report, polycythemia vera, JAK2 mutations, Ropeginterferon alfa-2b, young patient

## Abstract

This case report presents a 3-year-old female patient initially diagnosed with polycythemia vera (PV) in 2001. The patient exhibited elevated red blood cell (RBC) counts, high hemoglobin (Hb) levels, hyperleukocytosis, and moderate thrombocytosis, with sporadic abdominal pain and significant splenomegaly. Despite various treatments, including phlebotomies, hydroxyurea, and alpha-interferon, the patient struggled to maintain optimal hematocrit levels and experienced persistent symptoms. Subsequent genomic analysis revealed a rare *JAK2* G301R mutation alongside the canonical *JAK2* V617F mutation, potentially contributing to disease severity. In 2023, the patient started Ropeginterferon alfa-2b, leading to improved hematological parameters and symptom relief. The case underscores the challenges in managing PV, particularly in young patients, and highlights the potential clinical significance of additional *JAK2* mutations/variants and the potential benefits of Ropeginterferon alfa-2b in achieving better disease control.

## Introduction

Polycythemia vera (PV) is a rare myeloproliferative neoplasm (MPN) characterized by the overproduction of red blood cells (RBC) resulting in elevated RBC mass. There is often a concurrent stimulation of myeloid and megakaryocytic lineages, leading to increased white blood cell (WBC) and platelet (PLT) production. The *Janus kinase 2 (JAK2)* gain-of-function mutation V617F (exon 14) is associated with 97% of PV cases, with *JAK2* exon 12 mutations accounting for the remaining cases (1, 2). The introduction of next-generation sequencing (NGS) has provided a highly sensitive method for detecting other *JAK2* variants/mutations, though their clinical implications remain poorly understood (3). While the clinical course of PV in adults is relatively well-documented, its presentation and management in pediatric and young adult patients remain unclear, posing unique challenges.

Recent advances in PV treatment have introduced Ropeginterferon alfa-2b as a promising option, offering improved hematologic and molecular responses. This case report provides insight into the patient’s response to Ropeginterferon alfa-2b therapy, emphasizing its potential as a disease-modifying treatment for PV. Furthermore, the presence of multiple *JAK2* mutations, raises questions about their collective impact on disease progression and response to therapy. This case underscores the importance of continued research to elucidate the clinical significance of such mutations and optimize treatment strategies, particularly in young patients with PV.

Here, we present a case of a young PV patient carrying both the canonical V617F mutation and an exceedingly rare G301R mutation in the *JAK2* gene. Her disease has proven to be particularly challenging to control and less responsive to conventional therapies, creating significant management difficulties.

## Case presentation

In 2001, a 3-year-old female patient presented with polyglobulia (RBC 9,370,000/mmc), high hemoglobin (Hb) level (15.8 g/dl), and hematocrit (HCT) of 56.6%. Additionally, the patient exhibited hyperleukocytosis (WBC 28,670/mmc) and moderate thrombocytosis (PLT 679,000/mmc); the only reported symptom was sporadic abdominal pain. Physical examination revealed significant splenomegaly, with spleen’s lower pole reaching the transverse umbilical line.

The RBC mass was increased (54 ml/kg) and the investigation for endogenous erythroid colonies formation was positive, while the serum erythropoietin level was below the normal range (1.1 mU/ml). Respiratory function tests and airway resistances were within normal limits. BCR::ABL1 rearrangement and secondary causes of erythrocytosis were excluded. Histopathological analysis of bone marrow showed 95% cellularity, trilinear expansion, and abundant erythropoiesis. Megakaryocytes exhibited variable sizes and shapes (pleomorphic), ranging from small to large, either isolated or distributed in loose clusters; fibrosis grade was MF-0, with no evidence of blast cells. Cytogenetic analysis revealed a normal female karyotype in all metaphases analyzed. Therefore, a diagnosis of PV was made according to the 2001 World Health Organization criteria (4). Afterwards in 2005, PV diagnosis was confirmed by identifying the *JAK2* V617F mutation in 26% of the granulocytic population, via polymerase chain reaction (PCR) assay.

**Figure 1:**
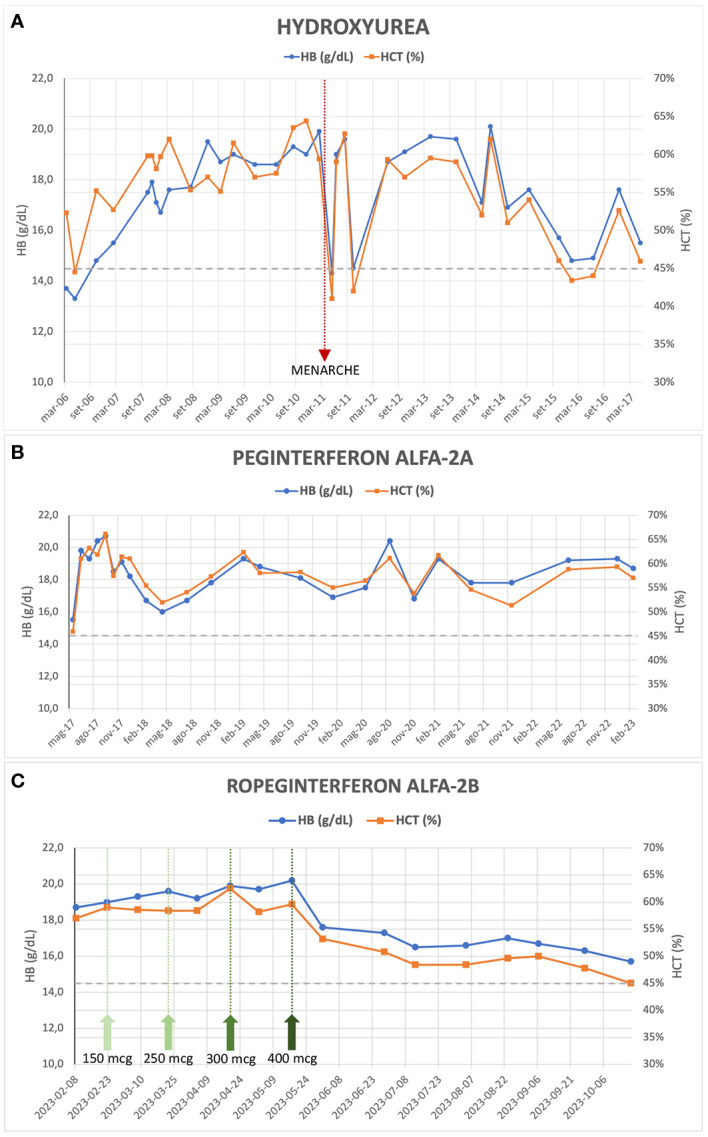
Evolution of hemoglobin and hematocrit values during treatment with Hydroxyurea **(A)**, Peginterferon alfa-2a **(B)** and Ropeginterferon alfa-2b **(C)**; the green arrows in **(C)** indicate the dose increments of the drug up to the maintenance dose of 400 mcg every two weeks.

The patient was initiated on antiplatelet therapy (ticlopidine 1/4 tablet of 250 mg per day) and underwent periodic phlebotomies from 2003 to 2006, with limited HCT control (HCT range 49.4 – 53.2%). An attempt to treat her with alfa-interferon (only one dose of 500,000 UI administered) in January 2006 was hindered due to severe side effects (fever and hypotension). Subsequently, from March 2006 to May 2017, the patient received hydroxyurea (HU) therapy and intermittent phlebotomies, considering the limited HCT control with phlebotomies alone and the significant microvascular symptoms of the disease despite ongoing antiplatelet therapy. This treatment has resulted in normalized WBC and PLT counts but inadequate HCT control (median HCT 57%, range 41 - 64.4%) and persistent symptoms (erythromelalgia, headache), along with splenomegaly (maximum longitudinal diameter 19-20 cm, approximately). Significant but temporary improvement in the patient’s blood count, reaching the HCT target of < 45%, was observed only in conjunction with the onset of menarche in April 2011 and the first menstrual cycles (**Figure 1A**). In November 2016, an NGS analysis was performed. The analysis confirmed the presence of the *JAK2* V617F mutation, with a variant allele frequency (VAF) of 21%, but identified an additional aminoacidic alteration in the gene (G301R, VAF 52%), previously unreported. In May 2017, the patient’s treatment was switched to Peginterferon alfa-2a (final dose 180 mcg/week) combined with phlebotomies when HCT values exceeded 60%. This high HCT threshold for performing phlebotomies was a consequence of the extreme difficulty in maintaining low HCT values without very frequent phlebotomies which, besides being impractical, were poor tolerated and often refused by the patient. However, satisfactory disease control was not achieved (median HCT 58%, range 46 – 66%) (**Figure 1B**).

During her clinical course, the patient underwent several reevaluations of bone marrow biopsy, which confirmed the presence of a chronic myeloproliferative neoplasm (MPN) consistent with PV, with unchanged fibrosis grade (MF-0). Furthermore, cytogenetic analysis confirmed the absence of alterations. In 2018, a repeated NGS analysis confirmed the presence of the double *JAK2* mutation (i.e., V617F and G301R, VAF 20% and 49%, respectively), without any further aberrations. Finally, in February 2023 at the age of 25 years, the patient started treatment with Ropeginterferon alfa-2b (starting dose 100 mcg every two weeks) and reached, after progressive dose escalation, the maximum maintenance dose of 400 mcg every two weeks by May 2023. After 8 months of follow-up, the patient demonstrated an improved response to this therapeutic regimen: currently, the patient does not exhibit any symptoms related to the hematological disease, although asymptomatic splenomegaly persists, with slight improvement (maximum longitudinal diameter of 18 cm on ultrasound). During Ropeginterferon therapy, the patient has never undergone phlebotomy treatment; Hb and HCT values have been showing a declining trend, and in the most recent blood count assessment (October 2023), the patient had an Hb level of 15.3 g/dL and an HCT of 45.0%, with WBC and PLT counts within the normal range (**Figure 1C**). The patient never experienced any thrombotic event nor other adverse events during treatment.

## Discussion

This case report describes a young patient with PV harboring both the *JAK2* V617F mutation and a rare G301R mutation. Canonical *JAK2* V617F and exon 12 mutations in MPNs are well described (1, 2). Several germlines or somatic *JAK2* variants were also described and some of them shown to confer hematopoietic cells cytokine independency or have been associated with gain-of-function catalytic activity (5). It is currently unknown whether such *JAK2* variants cooperate with V617F to provide a stronger gain of function over *JAK2* V617F alone but may be hypothesized that two aberrancies in *JAK2* may confer additive proliferative and/or survival advantages or induce genomic instability with higher mutagenic probability (6). Progression to acute myeloid leukemia (AML) from an MPN is typically associated with acquisition of genes mutation (e.g., *RUNX1, TP53, NRAS, WT1, FLT3, IDH1/2, TET2*) (7). In the recent era, *JAK2* variants were identified using NGS techniques, but, to our knowledge, only two previous studies (3, 8) reported the G301R mutation in 2 patients with MPNs, making it difficult to establish its clinical relevance. In a large retrospective study, Benton and colleagues reviewed the NGS data from 2154 patients affected by MPN, AML, or AML secondary to MPN, and found that 114 (5.3%) patients had a *JAK2* variant sequence identified, and 23 of these 114 patients had a concomitant *JAK2* V617F mutation (8). The presence of an additional *JAK2* variant was associated with a higher cumulative risk of progression to AML from MPNs, suggesting that the presence of *JAK2* V617F along with another *JAK2* variant may promote a more aggressive MPN with a higher transformative potential. The G301R mutation affects the FERM domain of the *JAK2* protein, responsible for the interaction with cytokine receptors (3). This could potentially have an impact on kinase activity and cell growth, with a subsequent increased myeloproliferative stimulus and a possible reduced responsiveness to treatments, although there is no evidence about it. Furthermore, it is important to emphasize that while some previously undescribed *JAK2* variants may represent mutations, other variants may merely represent polymorphisms (8); however, the G301R variant has not been previously reported in the Single Nucleotide Polymorphism Database (dbSNP). In the two aforementioned studies, it is not clarified whether the G301R mutation (identified with a VAF of 28% in one case (8) and 8% in the other (3)) can be considered a germline or somatic variant. In our patient, the G301R mutation had a VAF near 50% in both NGS analyses performed, and this suggests that it could represent a germline variant. Unfortunately, this hypothesis cannot be confirmed with certainty, as the presence of the G301R variant in other cell sources has not been studied.

The patient’s clinical course demonstrates the challenges in managing PV, especially when conventional therapies fail to achieve adequate control of HCT levels and symptoms. In adults, there are established guidelines for managing MPNs, which involve risk assessment, limiting cytoreductive treatments in case of high-risk categories. However, the same cannot be said for pediatric or young patients, as there is a limited amount of literature available. There are no cytoreductive agents approved for use in pediatric MPNs and the question of the leukemogenicity of HU remains controversial: there seems to be at most a slight increase in risk when HU is used alone (9), but this data is not confirmed by any large study. Consensus guidelines now recommend using interferon instead of HU as front-line therapy in younger patients (10). Most authors also agree that children with MPNs who are fully asymptomatic should not receive cytoreductive medications. In our patient, treatment with HU was started due to the presence of symptoms and to reduce the need for phlebotomy therapy, given the very high levels of Hb and HCT. Alfa-interferon not only restores normal blood cell counts in PV patients but can diminish the mutant *JAK2* V617F allele burden, which seems to be a risk factor for progression to secondary myelofibrosis (11). Results from PROUD-PV trial and its extension CONTINUATION-PV (12) led to regulatory approval of Ropeginterferon alfa-2b (BESREMi^®^), currently the only interferon approved in Europe for patients with PV and age ≥18, without symptomatic splenomegaly. The 6-year results of these trials (13) have shown an advantage of Ropeginterferon compared to the control arm (HU or best available treatment, with 88% of patients who received HU) in achieving complete hematologic responses (CHR, 54.5% versus 34.9%) and a significant increase in molecular responses (66% versus 19.4%), defined according to modified European LeukemiaNet (ELN) criteria (14). Interestingly, the patients treated with Ropeginterferon showed a more gradual onset of CHR compared with control-treated patients, but spent a higher proportion of time in CHR (13). The majority of Ropeginterferon alfa-2b-treated patients (54.3%) achieved a *JAK2* V617F allele burden <10% and might be potential candidates for treatment discontinuation. Furthermore, a five-fold lower incidence rate of disease progression (myelofibrosis or AML) was observed in Ropeginterferon-treated patients compared with the control arm, suggesting a disease-modifying potential of Ropeginterferon alfa-2b (15). The use of Ropeginterferon alfa-2b in the setting of patients with PV and low cardiovascular risk (young age and no previous thrombotic events) has already been evaluated in the phase 2 randomized LOW-PV trial, whose results have recently been published (16, 17). Ropeginterferon at a fixed dose of 100 mcg every 2 weeks demonstrated clear superiority in maintaining HCT levels on target if compared to phlebotomies alone (81.3% vs 50.8%, respectively, at 12 months; 82.7% vs 59.4%, respectively, at 24 months). It is noteworthy that in patients enrolled in the control arm who were non-responsive to phlebotomies and subsequently crossed over to Ropeginterferon, the drug proved to be less effective (17). In our patient, similarly unresponsive to phlebotomies, during the treatment with Ropeginterferon, a significant and consistent improvement in blood count values has been observed, achieving a CHR and reaching the target HCT level of 45% in the latest blood count assessment (**Figure 1C**). It is highly likely that the fixed low dose of 100 mcg administered every 2 weeks is not sufficient to improve treatment efficacy, and an escalated dose of Ropeginterferon, as used in the PROUD/CONTINUATION-PV study (12) and in our case, may be a more favorable approach to increase the proportion of patients achieving a CHR, even in cases of low cardiovascular risk. Despite the short follow-up period, our patient has well-tolerated the therapy so far, and no adverse effects have been recorded. The excellent tolerability of the drug, even in the case of dose escalation and maximum doses, is confirmed by comparable rates of discontinuation due to adverse events reported in the LOW-PV study (14%) and the PROUD/CONTINUATION-PV study (13%) (13, 17). The previous Peginterferon treatment does not alter the effectiveness of the drug, as also reported in other small series of patients (18).

In conclusion, the role of additional *JAK2* variants/mutations (e.g., G301R) in PV pathogenesis and its impact on disease progression and treatment response remain unclear and require further research. Given the potential increased risk of leukemic transformation and considering our patient’s young age, the long disease course, and the previous therapy with HU for more than 10 years, close clinical and disease monitoring is necessary to early detect any possible disease progression to myelofibrosis or AML. Interferon alfa remains one of the only treatments with the potential for disease modification and may reduce the risk of disease progression, given its impact on mutation burdens and induction of durable responses. A limitation of our case report is the lack of data on the monitoring of the patient’s *JAK2* V617F allele burden. Ropeginterferon alfa-2b treatment showed encouraging results in managing the disease, offering a potential therapeutic option for patients with difficult-to-control PV. Further studies are needed to better understand the prognostic impact of additional *JAK2* mutations and optimize treatment strategies for these patients.

## Data availability statement

The original contributions presented in the study are included in the article/supplementary material. Further inquiries can be directed to the corresponding author.

## Ethics statement

Ethical approval was not required for the study involving humans in accordance with the local legislation and institutional requirements. Written informed consent to participate in this study was not required from the participants or the participants’ legal guardians/next of kin in accordance with the national legislation and the institutional requirements. Written informed consent was obtained from the individual(s) for the publication of any potentially identifiable images or data included in this article.

## Author contributions

SL: Conceptualization, Investigation, Visualization, Writing – original draft. ES: Writing – original draft. IC: Writing – original draft. GP: Writing – original draft. MCM: Writing – original draft. RP: Writing – original draft. AC: Writing – original draft. MLB: Writing – original draft. MM: Writing – original draft. MB: Conceptualization, Supervision, Writing – review & editing.
